# Health economic analysis of seizure-related emergency visits of pediatric patients with drug-resistant epilepsy in Japan

**DOI:** 10.1016/j.ebr.2025.100840

**Published:** 2025-12-08

**Authors:** Ayataka Fujimoto, Takuya Kumagai, Kazuaki Yamamoto, Katsuhiko Iwasaki, Kinya Kokubo, Ataru Igarashi

**Affiliations:** a Epilepsy and Functional Neurosurgery, Department of Neurosurgery, Dokkyo Medical University School of Medicine, Tochigi, Japan; b Aculys Pharma, Inc., Tokyo, Japan; c Healthcare Consulting, Inc., Tokyo, Japan; d Faculty of International Political Science and Economics, Nishogakusha University, Tokyo, Japan; e Graduate School of Pharmaceutical Sciences, University of Tokyo, Tokyo, Japan

**Keywords:** Pediatric drug-resistant epilepsy, Health insurance claims database, Emergency transport costs, Total medical costs

## Abstract

This study estimates the cost of emergency transport and the total cost of medical care for pediatric patients with drug-resistant epilepsy (DRE) in Japan. We perform a retrospective analysis of patients aged less than 18 years with DRE. We use a health insurance claims database to identify the patients. The data are combined with published information, including government statistics and prior studies. We calculate the median medical cost for these patients after emergency and the cost per emergency transport service using recent data published by the Fire and Disaster Management Agency and calculation logic. The cost per emergency transport service is approximately 138,600 yen. Additionally, the cost of emergency transport for pediatric patients with DRE and the total medical cost in Japan are approximately 511,949,800 and 597,536,619 yen per year, respectively. This study is among the first to estimate the annual costs of seizure-related emergency transport and medical care for pediatric patients with DRE in Japan. These estimates can serve as baseline data for use in future assessments of the cost-effectiveness of acute medical care in this population.

## Highlights

• Estimated annual emergency transport and medical care costs for pediatric DRE in Japan.
• Estimated annual transport and medical care costs were 511,949,800 yen and 597,536,619 yen.
• Provides baseline estimates to support future cost-effectiveness assessments in DRE care.

## 1 Introduction

In Japan, epilepsy affects approximately 0.8 % of the population (1 million people) [1]. Advances in medicine, increased access to accurate diagnosis, and optimal antiseizure medications (ASMs) have enabled patients with epilepsy to become seizure-free and live a normal social life. However, 20 %–30 % of such patients fail to become seizure-free despite treatments [2]. Seizures that recur multiple times per day or those that last for minutes impose significant psychological, social, and economic burdens on both patients and caregivers [3,4].

Out-of-hospital use of anticonvulsants (rescue medications) can relieve seizures that are refractory to existing ASMs [[5], [6], [7], [8]]. Studies outside Japan examine the medical cost of seizure-related care and the health economic benefits of rescue medications [[9], [10], [11], [12]]. However, studies in Japan neither address the costs of seizure-related emergency transport or medical care in children or adolescents, nor the health economic benefits of out-of-hospital use of rescue medications for preventing emergency transport. Furthermore, Asano et al. [13] report that 95 % of patients transported to the hospital by ambulance for a second or subsequent seizure are considered less urgent or non-urgent, indicating that most emergency transports may be unnecessary. Thus, reducing these unnecessary emergency transports could lower the costs associated with emergency transportation and medical care. As a preliminary step toward evaluating whether out-of-hospital rescue medications can reduce the management costs of drug-resistant epilepsy (DRE), quantifying current costs is necessary. Given that only medications approved for pediatric use are covered by insurance in Japan, this study focuses on the pediatric population and estimates the annual costs of seizure-related emergency transport and medical care for pediatric patients with DRE. We aim to estimate the costs by patients’ age group.

## 2 Methods

### 2.1 Study design

This study performs a retrospective analysis of patients aged less than 18 years with DRE to estimate the cost of acute medical care for pediatric patients with epilepsy in Japan. We use a health insurance claims database to identify the patients. The data are integrated with published information, including government statistics and prior studies.

### 2.2 Study endpoints

The primary endpoints of the study are the cost of emergency transport and the total cost of outpatient and inpatient care following emergency department visits for pediatric patients with DRE. The secondary endpoints are the primary endpoints stratified by age group. The exploratory endpoints are the percentage of patients with DRE who visited the emergency department. The mean number of emergency visit cases per patient are compared between prefectures with and without epilepsy specialist medical facilities (i.e., epilepsy-support base hospitals designated by each prefecture or epilepsy centers that are member facilities of the Japan Epilepsy Center Association).

### 2.3 Data sources

The health insurance claims data are from the Cross Fact® insurer-based health insurance claims database [14,15]. As of August 2024, it contains data for 13.47 million individuals, covering 9.43 % of Japan’s 2023 total population [15]. The size of the Japanese population aged less than 18 years is based on population estimates of the Statistics Bureau of Japan [16]. Estimates of the prevalence of epilepsy and the proportion of those with epilepsy who have DRE are from Kurisu et al. [17] and Sultana et al. [18], respectively.

Using Sato’s [19] methods, the cost per emergency transport service is calculated using data from the Fire and Disaster Management Agency (FDMA) [20,21]. The cost per emergency transport is calculated by dividing the FDMA’s total expenditure by the number of staff, and then dividing that by the number of emergency transports per year. Healthcare price data, including health insurance points for medical services and Drug Price Standards, are retrieved from the Basic Master health insurance electronic claims-processing system (Medical Services Master, Pharmaceuticals Master, and Special Treatment Materials Master) of the Health Insurance Bureau of the Ministry of Health, Labor and Welfare [22].

### 2.4 Study population

The sample includes patients less than 18 years old who were diagnosed at least once under any of the International Statistical Classification of Diseases and Related Health Problems, Tenth Revision (ICD-10) codes G40.1–G40.9, G41.0–G41.2, G41.8, G41.9, R25.2, R56.0, or R56.8 (including uncertain diagnoses of epilepsy) between January 2018 and December 2022. Supplementary Table 1 lists the diseases and conditions corresponding to these ICD-10 codes.

The medical service (fee) codes listed in Supplementary Table 2 represent emergency visits based on a report from the Japan Health Insurance Association [23]. Supplementary Table 3 and 4 list the Japanese individual drug codes (Yakka Joho codes; YJ codes) and Anatomical Therapeutic Chemical codes for ASMs and anticonvulsants, respectively. ASMs are medications administered daily to patients for ongoing treatment, while anticonvulsants are medications used specifically during seizure episodes.

The patients in this study meet the following criteria: 1) diagnosed under an ICD-10 code of G40.1–G40.9, G41.0–G41.2, or G41.9 (i.e., documented diagnosis of epilepsy) between January 1, 2018, and December 31, 2022; less than 18 years old at the time of diagnosis; and prescribed at least 1 ASM during the month of diagnosis (i.e., pediatric patients with epilepsy); 2) with health insurance claims that reflected billing for any of the medical services listed in Supplementary Table 2 (i.e., visited an emergency department), and less than 18 years old on the date of billing for the service(s) (i.e., the “index date”); 3) diagnosed with epilepsy, as documented in the health insurance claim issued on the index date (i.e., documented diagnosis of epilepsy); 4) diagnosed with epilepsy within 6 months before the index date (excluding those whose first visit occurred on the index date); 5) prescribed at least 1 ASM at an outpatient department within 6 months before the index date; and 6) prescribed 2 or more ASMs during the entire period before the index date (i.e., patients with DRE as defined in this study). Patients with the health insurance claim billing for any of the medical services listed in Supplementary Table 2 between the day after admission and the day of discharge are excluded, as such patients could not have visited the emergency department on the index date.

### 2.5 Follow-up period

The baseline period is the time available for follow-up before the index date (up to 6 months). Patients who visited an emergency department on the index date and received only outpatient care before returning home are followed up for 1 day; those hospitalized after arriving at an emergency department are followed up until the day of discharge. The medical care provided during the follow-up period is analyzed.

### 2.6 Baseline clinical characteristics

Data on the clinical characteristics (diagnoses, rescue medications used, and ASMs used) of patients included in the analysis of claims data are collected for the baseline period. Sociodemographic data (age and sex) are collected as of the index date.

### 2.7 Data management

Healthcare price data are derived from the Basic Master health insurance claims-processing system for the years 2018, 2019, 2020, and 2022. The health insurance prices of individual medical services retrieved from the database are determined using medical service codes to link the price data from the Medical Services Master dataset to the medical services recorded in the database. Similarly, the health insurance prices of individual pharmaceuticals are determined by using YJ codes to link the price data from the Pharmaceuticals Master dataset to the use of pharmaceuticals recorded in the database. The health insurance prices of individual special treatment materials are determined by using special treatment material codes to link the price data from the Special Treatment Materials Master dataset to the use of special treatment materials recorded in the database.

### 2.8 Statistical analyses

The data for each continuous variable are presented as the number of patients, mean, standard deviation (SD), median, first quantile, and third quantile. Data on nominal variables are expressed as the number and percentage of patients in each stratum. Table 1 presents the logic used to estimate the emergency transport and total medical costs associated with emergency care for patients with DRE. Pediatric patients with DRE who visited the emergency department are categorized into 4 age groups (0–4, 5–9, 10–14, 15–17 years). The following parameters are calculated for each age group: percentage of patients visiting an emergency department, mean annual number of emergency visit cases per patient, percentage of emergency visit cases resulting in outpatient care, cost of outpatient care per emergency visit, percentage of emergency visit cases resulting in hospitalization, and cost of inpatient care per emergency visit. The percentage of pediatric patients with DRE visiting the emergency department and the mean annual number of emergency visit cases per patient are calculated as weighted means to account for the varying number of patients visiting the emergency department from 2018 to 2022. The costs of outpatient and inpatient care are determined by considering all medical services provided and all pharmaceuticals, and special treatment materials used to treat patients during the follow-up period.

**Table 1:** Summary of estimation logic for primary endpoints and associated variables

Variable	Details and definition			
Nonadult Japanese population by age group (×10^3^) --- [1]	0-4 years	5-9 years	10-14 years	15-17 years
	4,247	4,948	5,307	3,236
Prevalence of epilepsy (/1000 population) --- [2]	2.4	5.2	7.9	8.6
Estimated number of Japanese nonadult patients with epilepsy --- [3]	[1] × [2]			
Proportion of patients with drug-resistant epilepsy --- [4]	25%			
% of patients visiting the emergency department --- [5]	No. of patients with epilepsy who used emergency transport / total number of patients with epilepsy			
Average number of emergency visits per patient with emergency visit --- [6]	Cumulative number of emergency transports / No. of patients using emergency transport			
No. of emergency visits per patient with drug-resistant epilepsy --- [7]	[5] × [6]			
% of emergency visits with outpatient care --- [8]	No. of emergency visits with outpatient care / total number of emergency visits			
Cost of outpatient care per transport service (yen) --- [9]	Median cost of outpatient care per transport service			
% of emergency visits with hospitalization --- [10]	No. of emergency visits with hospitalization / total number of emergency visits			
Cost of inpatient care per hospitalization (yen) --- [11]	Median cost of inpatient care per hospitalization			
Endpoints:				
Emergency transport cost --- [15]	[3] × [4] × [7] × (cost per transport service)			
Cost of outpatient care following emergency visits --- [16]	[3] × [4] × [7] × [8] × [9]			
Cost of inpatient care following emergency visits --- [17]	[3] × [4] × [7] × [10] × [11]			

The number of pediatric patients with DRE in Japan is estimated by multiplying the population statistics for 2022 by the previously estimated prevalence of epilepsy [17] and the percentage of patients with epilepsy who had DRE (25 %) [18]. Using this number and the median costs of outpatient and inpatient care (obtained from claims analysis, the total annual medical cost associated with emergency visits and care of pediatric patients with DRE is estimated for both the entire population and each age group. Following Sato’s [19] method, the cost per transport service is estimated from data published by the FDMA. This cost is used to estimate the annual cost of emergency transport for pediatric patients with DRE.

We compare the percentage of pediatric patients with epilepsy who visited the emergency department and the mean number of emergency visit cases per patient between prefectures with and without epilepsy specialist medical facilities (Supplementary Table 5). The presence or absence of such specialty facilities is based on the registration status of facilities in 2024. For each such patient, we count the number of emergency visits that occurred during the follow-up period. We calculate the percentage of those patients who visited the emergency department at least once. The total number of emergency visit cases was divided by the cumulative duration of follow-up (in years) to calculate the average number of emergency visit cases per year. Data are analyzed using the Fisher exact test (percentage of patients visiting the emergency department) or the Wilcoxon rank sum test (average number of emergency visit cases per year). A *P* value < 0.05 is considered statistically significant. Statistical analyses are performed using R software, version 4.3.1 (R Foundation for Statistical Computing).

## 3 Results

### 3.1 Patient characteristics at baseline and Outcomes of interest

**Fig. 1:**
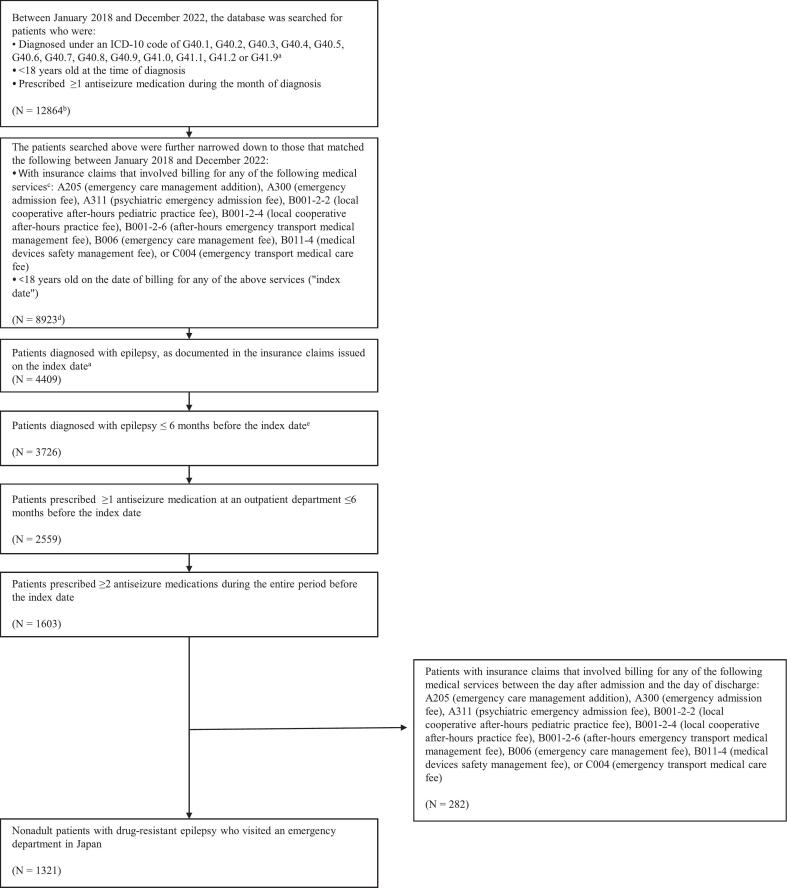
Flowchart for selection of the study population. ICD-10, International Statistical Classification of Diseases and Related Health Problems, Tenth Revision. ^a^ Patients with a documented diagnosis of epilepsy. ^b^ Number of unique patients. ^c^ Patients receiving any of the services were regarded as visiting an emergency department. ^d^ A single patient visiting > 1 emergency department was counted each time. ^e^ Excluding patients whose first visit was on the index date.

Fig. 1 illustrates how the patients are selected. A total of 1321 pediatric patients with DRE are included in the health insurance claims database. Table 2 presents the baseline characteristics of this patient population. Of the patients included in the analysis of claims data, only 6.7 % are prescribed some anticonvulsants at an ambulance-receiving hospital on the date of emergency visits (index date). Of the patients who visited the emergency department, 54.0 % are hospitalized on the date of transportation or the following day, and 50.6 % are prescribed no anticonvulsants on the date of transportation but are hospitalized on that date or the next day.

**Table 2:** Demographic and clinical characteristics of patients at baseline

	Nonadult patients with drug-resistant epilepsy who visited the emergency department (N = 1321)
Age (years)	
Mean (SD)	8.0 (5.1)
0-4, n (%)	459 (34.7)
5- 9, n (%)	329 (24.9)
10-14, n (%)	346 (26.2)
15-17, n (%)	187 (14.2)
Sex	
Male, n (%)	753 (57.0)
Anticonvulsant prescribed at the ambulance-receiving hospital on the index date	
Yes, n (%)	89 (6.7)
Anticonvulsants prescribed at the ambulance-receiving hospital on the index date^a^	
Diazepam, n (%)	37 (41.6)
Midazolam, n (%)	30 (33.7)
Thiamylal sodium, n (%)	2 (2.2)
Thiopental sodium, n (%)	4 (4.5)
Phenobarbital, n (%)	7 (7.9)
Phenobarbital sodium, n (%)	7 (7.9)
Chloral hydrate, n (%)	1 (1.1)
Phenytoin sodium, n (%)	1 (1.1)
Fosphenytoin sodium hydrate, n (%)	13 (14.6)
Levetiracetam, n (%)	18 (20.2)
Hospitalization on the index date or the following day	
Yes, n (%)	713 (54.0)
Duration of hospitalization (days)	
Median (interquartile range)	6 (3-11)
Anticonvulsant prescribed on the index date and hospitalization on the index date or the following day	
Prescribing any anticonvulsant and hospitalized, n (%)	44 (3.3)
Prescribing any anticonvulsant but not hospitalized, n (%)	45 (3.4)
Not prescribing any anticonvulsant but hospitalized, n (%)	669 (50.6)
Not prescribing any anticonvulsant and not hospitalized, n (%)	563 (42.6)
Diagnosis (ICD-10 codes) made during the baseline period (by the month before the index date)	
G40.1. Localization-related (focal) (partial) symptomatic epilepsy and epileptic syndromes with simple partial seizures, n (%)	49 (3.7)
G40.2. Localization-related (focal) (partial) symptomatic epilepsy and epileptic syndromes with complex partial seizures, n (%)	154 (11.7)
G40.3. Generalized idiopathic epilepsy and epileptic syndromes, n (%)	98 (7.4)
G40.4. Other generalized epilepsy and epileptic syndromes, n (%)	425 (32.2)
G40.5. Special epileptic syndromes, n (%)	0 (0)
G40.6. Grand mal seizures, unspecified (with or without petit mal), n (%)	11 (0.8)
G40.7. Petit mal seizures, unspecified, without grand mal seizures, n (%)	47 (3.6)
G40.8. Other epilepsy, n (%)	596 (45.1)
G40.9. Epilepsy, unspecified, n (%)	1126 (85.2)
G41.0. Grand mal status epilepticus, n (%)	17 (1.3)
G41.1. Petit mal status epilepticus, n (%)	1 (0.1)
G41.2. Complex partial status epilepticus, n (%)	10 (0.8)
G41.9. Status epilepticus, unspecified, n (%)	178 (13.5)
No. of antiseizure medications taken before the index date	
Mean (SD)	3.3 (1.9)
No. of antiseizure medications taken before the index date	
2, n (%)	596 (45.0)
3, n (%)	338 (25.6)
4, n (%)	169 (12.8)
5, n (%)	101 (7.6)
6, n (%)	60 (4.5)
7, n (%)	11 (0.8)
8, n (%)	18 (1.4)
9, n (%)	6 (0.5)
≥10, n (%)	23 (1.7)
Home rescue medication prescribed before the index date	
Yes, n (%)	710 (53.7)
Home rescue medications prescribed before the index date^b^	
Diazepam (suppository), n (%)	676 (95.2)
Midazolam (oral solution), n (%)	52 (7.3)
Phenobarbital sodium (suppository), n (%)	31 (4.4)
Chloral hydrate (suppository), n (%)	70 (9.9)
Chloral hydrate (rectal kit), n (%)	62 (8.7)

Abbreviations: SD, standard deviation, ICD-10, International Statistical Classification of Diseases and Related Health Problems, Tenth Revision.

a The number of patients prescribed any rescue medication at the ambulance-receiving hospital on the index date was used as the denominator to calculate the percentages.

b The number of patients prescribed any home rescue medication during the period before the index date was used as the denominator to calculate the percentages.

### 3.2 Analysis of health insurance claims data

Table 3 presents data on several parameters, stratified by the 4 patient-age groups.Table 4.

**Table 3:** Primary study variables and outcomes of interest, by patient age group

	0-4 years	5-9 years	10-14 years	15-17 years
% of patients visiting the emergency department				
% (n/N)*	20.3 (208/1024)	9.3 (214/2313)	4.6 (148/3249)	5.5 (139/2518)
No. of emergency visit cases per patient per year				
Mean (n/N)	2.2 (459/208)	1.5 (329/214)	2.3 (346/148)	1.3 (187/139)
% of emergency visit cases resulting in outpatient care				
% (n/N)	48.8 (224/459)	59.9 (197/329)	62.7 (217/346)	53.5 (100/187)
Cost of outpatient care per emergency visit (yen)				
Median (Q1-Q3)	13,493.75 (10,635.00-26,256.245)	20,443.20 (9,340.00-45,603.00)	124,460.00 (26,054.29-213,530.00)	31,200.00 (11,145.05-64,097.38)
% Emergency visit cases resulting in hospitalization				
% (n/N)	60.6 (278/459)	47.7 (157/329)	46.0 (159/346)	63.6 (119/187)
Cost of inpatient care per emergency visit (yen)				
Median (Q1-Q3)	277,534.00 (170,727.42-552,401.12)	232,563.50 (121,540.00-458,655.40)	228,489.00 (77,389.37-502,552.76)	216,588.78 (68,815.00-509,715.75)

Abbreviations: ICD-10, International Statistical Classification of Diseases and Related Health Problems, Tenth Revision; Q1, first quartile; Q3, third quartile.

*N here denotes the number of unique epilepsy patients who were diagnosed under an ICD-10 code of G40.1, G40.2, G40.3, G40.4, G40.5, G40.6, G40.7, G40.9, G41.0, G41.1, G41.2, or G41.9 between January 2018 and December 2022 AND were <18 years old at the time of diagnosis AND were given ≥1 antiepileptic drug during the month of diagnosis, multiplied by the percentage of patients with drug-resistant epilepsy (25%).

**Table 4:** Estimated emergency transport cost and total medical cost among nonadult Japanese patients with drug-resistant epilepsy

	Age groups				Overall
	0-4 years	5-9 years	10-14 years	15-17 years	0-17 years
Emergency transport cost (yen)	15,84,19,800	12,69,57,600	15,48,16,200	7,16,56,200	51,18,49,800
Total medical cost per year (yen)	19,98,60,575	11,30,86,130	20,46,89,806	7,99,00,109	59,75,36,619
Annual cost of outpatient care following emergency visit (yen)	75,29,513	1,12,23,317	8,72,46,460	86,42,400	11,46,41,689
Annual cost of inpatient care following emergency visit (yen)	19,23,31,062	10,18,62,813	11,74,43,346	7,12,57,709	48,28,94,930

### 3.3 Estimated number of pediatric patients with DRE

Based on epidemiological data [17], the estimated numbers of pediatric patients with DRE in Japan are as follows: 2,549 patients (0 to 4 years old), 6,433 patients (5 to 9 years old), 10,482 patients (10 to 14 years old), and 6958 patients (15 to 17 years old).

### 3.4 Cost per emergency transport service

The cost per emergency transport service is 138,600 yen.

### 3.5 Costs of seizure-related emergency transport and medical care for pediatric patients with DRE

Table 4 presents the estimated seizure-related medical and nonmedical (i.e., transport service per se) costs for pediatric patients with DRE. For the entire study population (0–17 years old), the total annual cost of emergency transport services is 511,849,800 yen, and the total annual medical cost is 597,536,619 yen. Supplementary Table 6 shows the costs for the three scenarios— “all patients received emergency medical services but were not transported,” “all patients received only outpatient care and returned home after emergency transport,” and “all patients were hospitalized after emergency transport”.

### 3.6 Emergency department visits in prefectures with vs without epilepsy specialist medical facilities

The percentage of patients visiting an emergency department is similar between prefectures with and without epilepsy specialty medical facilities (8.05 % [680/8449] vs 7.16 % [122/1703]; *P* > 0.05). The mean and median annual numbers of emergency visit cases per patient are also similar between these 2 categories of prefectures (mean [SD], 0.74 [0.99] vs 1.02 [1.86]; median [interquartile range (IQR)], 0.40 [0.23–0.75] vs 0.29 [0.21–0.80]; *P* > 0.05).

## 4 Discussion

### 4.1 Baseline characteristics of patients

Of the patients included in the analysis of claims data, 93.3 % are not prescribed an anticonvulsant at an ambulance-receiving hospital on the date of emergency visit (index date), probably because their seizures have been resolved on hospital arrival. Of the patients who visited the emergency department, 46.0 % presumably have had their care completed in the department. However, 50.6 % of patients who visited the emergency department have not been prescribed any anticonvulsant on the date of the visit but have been hospitalized on that or the next day. These results indicate that approximately half of the patients visiting the emergency department require inpatient care, even if their seizures are resolved by the time they arrive at the hospital.

### 4.2 Emergency transport cost

The Tokyo Fire Department states that the cost per emergency transport service is 45,000 yen [24]. However, this estimate likely fails to reflect current prices and is not generalizable nationwide. Using the 2010 FDMA financial report, Sato [19] estimates that the cost of each emergency transport is 120,800 yen; however, this estimate is still not consistent with current prices. Following Sato’s [19] method and using the 2020 FDMA financial report, we estimate the cost per emergency transport service to be 138,600 yen.

### 4.3 Percentage of patients visiting an emergency department and number of emergency visit cases

Approximately 13 % to 17 % of patients with epilepsy have seizure-related visits to the emergency department [25,26]. According to a survey of caregivers for children with epilepsy in Japan, 28.4 % of children with DRE use emergency transport [8]. In this study, we estimate that the proportion of pediatric patients with DRE who visited the emergency department ranged from 4.6 % to 20.3 %, depending on age, consistent with the previous findings.

In pediatric epilepsy, high rates of emergency department visits are partly attributed to the elevated anxiety commonly experienced by caregivers [27], which is considered a factor promoting emergency department utilization during seizures [28]. The increased emergency department visits observed in this study may be due to caregiver anxiety, supporting existing studies. However, more frequent emergency department visits may also be due to incorrect or absent administration of emergency seizure medication [29], suggesting that such suboptimal use may have occurred in some cases. Other possible factors include physician instructions to arrange emergency transport after seizures and caregiver perceptions that certain rescue medications require immediate transport following administration.

### 4.4 Estimated costs of Seizure-Related emergency transport and medical care

In the United Kingdom, the total annual cost of emergency transport of patients with convulsions or seizures (including those not eventually transported to hospitals) is 113,880,194 yen (based on a 2011 exchange rate of £1 to 127.9340 yen), and 64.4 % of this total cost (73,304,007 yen, per the same exchange rate) pertain to care for patients transported to hospitals [9]. In this study, the annual cost of emergency transport for pediatric patients with DRE in Japan is based on the number of patients driven to hospitals; patients who are not ultimately transported to hospitals are excluded. Thus, the inclusion of costs associated with ambulance dispatches that do not involve transporting patients to hospitals would have resulted in a greater estimated total cost of emergency transport for pediatric patients with DRE, as reported in this study.

According to extant literature, of the total cost of medical care for patients with epilepsy, less than 50 % is spent on the management of the condition, and a greater percentage of the overall cost is associated with care for comorbidities [30,31]. In this study, the total medical cost includes costs associated with treatment for comorbidities. The results show that median costs of post-transport care are between 13,493.75 and 124,460.00 yen for outpatient care and between 216,588.78 and 277,534.00 yen for inpatient care. Estimated outpatient care is more expensive for those aged 10–14 years than for other age groups primarily due to the inclusion of artificial respirators in some cases (64,800 yen). Many 10–14-year-old patients who required ventilator management during the period are included in the database; these patients require home ventilator management for diseases such as congenital diseases and cerebral palsy. Seven unique patients (94 emergency visits) who required home ventilator management are eligible, and four of them (66 emergency visits) are aged 10 to 14 years, accounting for 70.2 % of the emergency visits frequency of patients requiring ventilator management. Thus, we deduce that the medical expenses for patients aged 10 to 14 years are higher than for other age groups. Borghs et al. [10] examine the costs of seizure-related medical care for patients with epilepsy who had private insurance. They estimate that the median (IQR) cost of emergency department visits is 211,240 (46,046–459,692) yen and the median (IQR) cost of inpatient care is 2,462,989 (1,583,027–3,985,836) yen, based on a 2018 exchange rate of $1.00 to 110.4232 yen. As such, our results do not notably differ from those of existing studies; nevertheless, intercountry variations in costs of medical services and drugs may preclude direct comparisons.

### 4.5 Potential cost savings due to preventing emergency visit cases

Okazaki et al. [8] showed that the prompt use of rescue medications for seizures occurring outside a hospital setting may significantly shorten the time to seizure resolution and reduce the likelihood of emergency transport. Based on this evidence, we expect that appropriate use of rescue medications for out-of-hospital seizures can help prevent emergency transport and consequently reduce seizure-related emergency transport and total medical costs. Existing studies focusing on the United States suggest that out-of-hospital use of rescue medications may reduce emergency department visits by 50 % to 71 % [25,32,33]. This reduction can realize savings of 255,924,900 to 363,413,358 yen in emergency transport costs and 298,768,309 to 424,250,999 yen in total medical costs. Greater cost savings might be possible if rescue medications could resolve a higher percentage of out-of-hospital seizures. Rescue medications approved for use in Japan include suppositories of diazepam, chloral hydrate, and phenobarbital sodium, as well as chloral hydrate rectal kits and midazolam oral solution; the health insurance prices for these drugs at their central doses range from 40.5 to 2966.7 yen per drug. In this study, we estimate 3693 emergency visit cases of pediatric patients with DRE in Japan per year. If rescue medications are used to resolve seizures in such patients before emergency visit, the total cost of these rescue medications would be between 149,567 to 10,956,023 yen per year, which is less than the cost of emergency transport and medical care in emergency visit cases.

As rescue medications remain underused for epileptic seizures [26], promoting and optimizing the use of these drugs would prevent seizure-related visits to emergency departments. It can thus result in reduced emergency transport costs and total medical costs associated with DRE. Nunley et al. [34] report significantly fewer emergency department visits and lower rates of ambulance utilization among patients who received medications in a different manner, receiving rescue medications prescribed intranasally compared to those receiving rectal medications. This suggests a greater efficacy of intranasal rescue medications for preventing seizure-related emergency visits.

Developing seizure-action plans (SAPs) or acute seizure-action plans (ASAPs) is also important to reduce both the cost of emergency transport and total medical costs associated with seizure-related emergency care. A survey finds that only 20 % of patients use rescue medications for immediate treatment of seizure clusters, even though 79 % of clinicians recommend the use of these drugs [3]; the inconsistent data suggest that establishing SAPs/ASAPs can facilitate the use of rescue medications. ASAPs that recommend the use of rescue medications may reduce the overutilization of emergency care, thereby generating savings in both the direct and indirect costs of epileptic seizures [35]. SAPs/ASAPs should be developed to improve seizure control and patient quality of life, as well as to optimize the utilization of medical resources such as seizure-related emergency visits and care. The study’s results highlight the need to find ways to improve medical care and support for pediatric patients with DRE and optimize management for those patients to conserve medical resources.

### 4.6 Frequency and number of emergency visit cases in prefectures with vs without epilepsy specialist medical facilities

Prefectures with medical facilities providing specialty care for epilepsy can reduce the number of seizure-related emergency visit cases by implementing better patient education for seizure control and stricter criteria for seizure-related emergency visit. Our study reveals that the percentage of patients visiting the emergency department and the mean annual number of emergency visit cases per patient do not differ significantly between prefectures with and without epilepsy specialist medical facilities, although, prefectures with such facilities have a smaller mean annual number of emergency visit cases per patient. The failure to detect any regional differences may partly be due to the incomplete overlap between administrative areas and medical zones. That is, a hospital providing epilepsy support services or an epilepsy center in an administrative area may not offer complete care for all patients with epilepsy who live in the area. Future studies should compare the number of emergency visits per patient among individuals treated at epilepsy specialist medical facilities to those treated elsewhere.

### 4.7 Limitations of the study

This study has limitations. First, the percentage of patients with DRE that is used in the estimations is based on the estimate of Sultana et al. [18], which may not reflect the true proportion of patients with epilepsy who have DRE in Japan.

Second, the health insurance claims database lacks data on how patients arrive at hospitals. Therefore, we used medical services usually billed along with an emergency visit case to identify pediatric patients with DRE visiting emergency departments. However, some of these services may also be billed for patients who visit an emergency department on foot or by private vehicle. Although the diagnosis of epilepsy shown in Supplementary Table 1 is recorded at the emergency visit, this visit by pediatric patients with epilepsy may be for reasons other than epilepsy. In short, the medical expenses could include medical treatment for diseases and symptoms other than seizures.

Third, the Consensus Proposal by the International League Against Epilepsy (ILAE) [36] and the Japanese Society of Neurology (JSN) Clinical Practice Guidelines for Epilepsy 2018 [37] define DRE as “failure of adequate trials of two tolerated, appropriately chosen and used ASM schedules (whether as monotherapies or in combination) to achieve sustained seizure freedom.” In this study, we define patients treated with at least 2 ASMs during the available period before the index date (date of emergency visit) as having DRE. As we lack access to data regarding medical care received before the period covered by the claims database, our study population may have excluded patients with DRE as defined by the ILAE or JSN guidelines or may have included patients who do not meet this definition of DRE. Given that the nationwide Cross Fact® claims database has no known geographic or age biases, we believe that the patients with DRE who are identified from this database represent all such patients in Japan.

Fourth, data on emergency transport costs are not available either on the FDMA website or in the white papers prepared by this agency. To obtain a rough estimate of emergency transport costs, we use the settled cost (prorated by the number of employees) on the 2020 FDMA financial report; however, this estimate may differ considerably from the true cost. Additionally, the number of ambulance dispatches and paramedics assigned to pre-hospital emergency care vary greatly by region.

Fifth, the scope of the research could be expanded to include more cases to verify reproducibility. The database used in this study includes many patients aged 10 to 14 years who required ventilator management. Medical expenses for patients aged 10 to 14 are higher than for other age groups due to the impact of patients requiring ventilator management. Concerns remain regarding the representativeness of medical expenses for this age group, and further investigation is necessary.

Sixth, we could not confirm the occurrence of out-of-hospital use of rescue medications based on the health insurance claims data for the patients included in our analysis. Therefore, the study population includes patients who were treated with rescue medication prior to arriving at a hospital. Future studies can evaluate the extent to which emergency transport can be avoided in Japan through the use of rescue medication and other methods. The percentage of seizure-related emergency visit cases that can be prevented by out-of-hospital use of rescue medications may vary with the type of rescue medication. To clarify the role of out-of-hospital use of rescue medications in seizure control, future studies can compare patients who have been educated about SAP/ASAP-based, out-of-hospital care, including the use of rescue medication, with patients treated according to current strategies.

Seventh, we could not quantify statistical uncertainty about the costs (e.g., confidence or uncertainty intervals). We derived our estimates by deterministically combining data from claims databases, public epidemiologic statistics, and reports issued by public institutions. Because patient-level linkages and variance–covariance information across these sources were unavailable, constructing valid intervals would have required unverifiable assumptions.

Lastly, our study period is from January 2018 to December 2022. Therefore, medical facilities that have been registered as either hospitals providing epilepsy support services or epilepsy centers between 2022 and 2024 have been excluded from this study. However, the exclusion of such facilities may not have greatly impacted our results since these facilities would likely have had functions that made them nearly qualified as epilepsy specialist medical facilities during the search period.

## 5 Conclusions

This study is among the first to estimate the annual costs of seizure-related emergency transport and medical care for pediatric patients with DRE in Japan. If optimal out-of-hospital use of rescue medications for seizure control could prevent emergency visits of such patients, this practice would yield savings in both the cost of emergency transport and total medical costs associated with DRE. These cost savings would increase as the percentage of seizures that could be controlled with rescue medications grow. The estimates in this study serve as baseline data for use in future assessments of the cost-effectiveness of rescue medications.

## Author Contributions

AF supervised the study, interpreted the data, and revised the manuscript. TK conceptualized, designed, and supervised the study, obtained study funding, interpreted the data, oversaw the project, and revised the manuscript. KY acquired and managed the data, managed the project, created programs to clean and prepare the data for analysis, performed the statistical analysis, created the figures, and wrote and revised the manuscript. KI interpreted the data and revised the manuscript. KK designed the study and revised the manuscript. AI conceptualized, designed, and supervised the study, interpreted the data, and revised the manuscript. All authors critically reviewed and approved the manuscript.

## Ethical Statement

This study’s protocol has been reviewed and approved by the Ethics Committee of the Japanese Association for the Promotion of State-of-the-Art in Medicine (approval number: JR40). All patient data contained in the health insurance claims database have been anonymized, and no personal information has been collected. Thus, informed consent is not required.

## CRediT authorship contribution statement

**Ayataka Fujimoto:** Writing – review & editing, Validation, Supervision. **Takuya Kumagai:** Writing – review & editing, Validation, Supervision, Resources, Project administration, Methodology, Funding acquisition, Conceptualization. **Kazuaki Yamamoto:** Writing – original draft, Visualization, Software, Project administration, Methodology, Investigation, Formal analysis, Data curation. **Katsuhiko Iwasaki:** Writing – review & editing, Validation. **Kinya Kokubo:** Writing – review & editing, Methodology, Conceptualization. **Ataru Igarashi:** Writing – review & editing, Validation, Supervision, Methodology, Conceptualization.

## Funding

This study was funded by Aculys Pharma, Inc.

## Declaration of competing interest

The authors declare the following financial interests/personal relationships which may be considered as potential competing interests: AF received honoraria for lectures and advisory fees from Aculys Pharma, Inc. TK is an employee of Aculys Pharma, Inc. Healthcare Consulting, Inc. has received research funding from Aculys Pharma, Inc. KK is chief executive officer at Healthcare Consulting, Inc. KI and KY are employees of Healthcare Consulting, Inc. AI received advisory fees from Aculys Pharma, Inc. for this work. There are no other conflicts of interest to declare.
